# Teetering on the Edge: Second-Degree Atrioventricular Block Following Long-Acting Second-Generation Antipsychotic

**DOI:** 10.7759/cureus.36650

**Published:** 2023-03-24

**Authors:** Kamahl Harrisingh, Cameron Cu, Marjolein Le Le, Daniel Benhayon

**Affiliations:** 1 Internal Medicine, Memorial Healthcare System, Hollywood, USA; 2 Cardiology, Memorial Healthcare, Pembroke Pines, USA; 3 Electrophysiology, Memorial Healthcare, Pembroke Pines, USA

**Keywords:** drug-induced arrhythmia, second generation antipsychotics, paliperidone, long acting injectable, atrio-ventricular block, drug-related side effects and adverse reactions

## Abstract

Our case report highlights the importance of understanding various mechanisms of an atrioventricular block (AVB) and recognizing potential iatrogenic culprits. Despite the prevalent use of second-generation antipsychotics and the growing popularity of long-acting formulations, it is not routinely recognized as a cause for AVB. Second-generation antipsychotics such as risperidone have a dose-dependent pro-arrhythmic effect and are known to cause first-degree AVB. Our case presents an opportunity to recognize an unappreciated cause for AVB and switch to safer alternatives. In the era of long-acting injectables, it is important to monitor for these effects prior to escalating doses and risking high-degree AVB.

## Introduction

While non-adherence to medications is seen across all disciplines of medicine, it is particularly common in psychiatric patients due to the lack of awareness of their illness (anosognosia). For such reasons, long-acting psychotropic formulations, with high-loading doses are often favored. Herein, we present a case of a 31-year-old male who presented with a new onset second-degree atrioventricular block (AVB) following a recent loading dose of a long-acting second-generation antipsychotic.

## Case presentation

**Figure 1:**
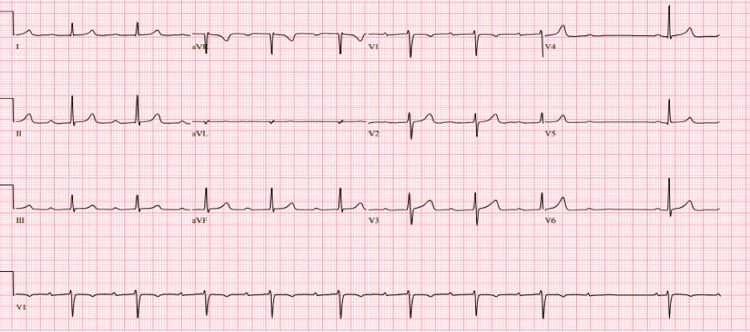
EKG on admission showing second-degree Mobitz Type 1 AV block

**Figure 2:**
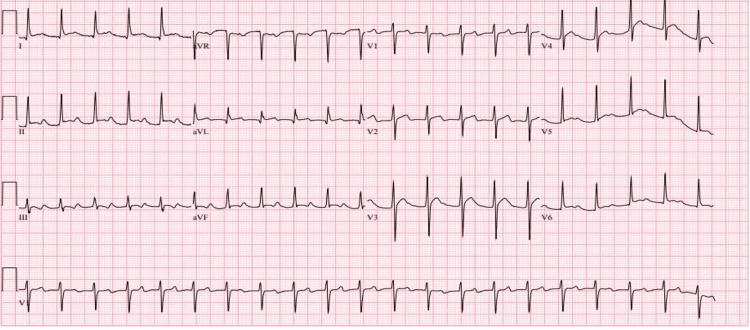
EKG 1 year prior to admission showing sinus tachycardia with a PR interval of 272

A 31-year-old African American male with schizophrenia, hyperthyroidism, and marijuana use disorder presented with complaints of auditory hallucinations and intermittent headaches, which had been ongoing for the past week. He denied any fever, chills, dyspnea, chest pain, palpitations, or feeling of missed beats. Physical exam revealed an anxious affect but otherwise unremarkable. The metabolic panel and complete blood count were within normal limits. Ethanol levels were less than 10mg/ml, Urine drug screen was positive for cannabinoids, and his TSH levels were 2.3mIU/L. Telemetry and electrocardiogram (ECG) showed new onset second-degree Mobitz type 1 AVB (Figure 1). He had ECGs while on oral Risperidone only showing first-degree AVB (Figure 2), and a normal echocardiogram two years prior. He was not taking any well-known atrioventricular nodal blocking agents, and had no prior cardiac conditions, recent infections, or familial cardiac disease. A detailed review of his medications revealed a recent loading dose of 234 mg Invega Sustenna, a second-generation antipsychotic. He also continued intermittent use of 3mg oral Risperidone. He was hemodynamically stable and did not require urgent intervention. The recommendation was made to discontinue Risperidone along with future Invega injections in light of suitable alternatives and close follow-up with repeat EKGs. Patient was unfortunately lost to follow up. 

## Discussion

Common reversible causes of AVB were evaluated. He had no ischemic heart disease, cardiomyopathy, or myocarditis. TSH and electrolytes were within the normal range. His recent initiation on Invega Sustenna, a second-generation antipsychotic containing the active ingredient Paliperidone combined with palmitate to provide a long-acting formulation was of concern [1]. He also continued intermittent use of 3mg oral Risperidone, which also contains the active metabolite Paliperidone. There have been some case reports linking Paliperidone to first-degree AV blocks, but no documented cases with second-degree AV block [2-3]. Paliperidone has a known dose-dependent pro-arrhythmic effect [4]. The high initial loading doses may increase the risk of higher degree AV blocks. We suspect that the combination of second-generation antipsychotics, particularly in the high doses used for loading, potentiated the class effect of AVB. 

## Conclusions

Our case and a few others have shown that second-generation antipsychotics can be associated with AVB. Our case highlights a concerning risk of higher degree AVB with increased doses. This presents an opportunity to recognize a potential unappreciated cause for AVB, and switch to safer alternatives before further progression. In an era of long-acting injectables, it is important to monitor for these effects prior to initiating or escalating doses. Concurrent use of long-acting and short-acting formulations of Paliperidone, or with other pro-arrhythmic agents should be done with caution.

## References

[1] (2012). LiverTox: Clinical and Research Information on Drug-Induced Liver Injury [Internet]. Bethesda (MD). https://www.ncbi.nlm.nih.gov/books/NBK548506/.

[2] Cheung B, Levy C, Shivkumar A (2020). First-degree atrioventricular block with tachycardia from Paliperidone and Mirtazapine overdose. Eur J Case Rep Intern Med.

[3] Jegede O, Taher K (2019). Risperidone-associated atrioventricular block. Am J Ther.

[4] Yasin M, Lateef N, Pervaiz MH (2018). Paliperidone-associated atrioventricular block. Am J Ther.

